# Sex and age differences in social and cognitive function in offspring exposed to late gestational hypoxia

**DOI:** 10.21203/rs.3.rs-2507737/v1

**Published:** 2023-06-07

**Authors:** Steve Mabry, E. Nicole Wilson, Jessica L. Bradshaw, Jennifer J. Gardner, Oluwadarasimi Fadeyibi, Edward Vera, Oluwatobiloba Osikoya, Spencer C. Cushen, Dimitrios Karamichos, Styliani Goulopoulou, Rebecca L. Cunningham

**Affiliations:** UNTHSC: University of North Texas Health Science Center; UNTHSC: University of North Texas Health Science Center; UNTHSC: University of North Texas Health Science Center; UNTHSC: University of North Texas Health Science Center; UNTHSC: University of North Texas Health Science Center; UNTHSC: University of North Texas Health Science Center; UNTHSC: University of North Texas Health Science Center; UNTHSC: University of North Texas Health Science Center; UNTHSC: University of North Texas Health Science Center; Loma Linda University School of Medicine; UNT Health Science Center: University of North Texas Health Science Center

**Keywords:** prenatal programming, sex differences, chronic intermittent hypoxia, marble burying behaviors, open field, social behaviors, Morris water maze, hippocampus, autism spectrum disorder

## Abstract

**Background:**

Gestational sleep apnea affects 8-26% of pregnancies and can increase the risk for autism spectrum disorder (ASD) in offspring. ASD is a neurodevelopmental disorder associated with social dysfunction, repetitive behaviors, anxiety, and cognitive impairment. To examine the relationship between gestational sleep apnea and ASD-associated behaviors, we used a chronic intermittent hypoxia (CIH) protocol between gestational days (GD) 15-19 in pregnant rats to model late gestational sleep apnea. We hypothesized that late gestational CIH would produce sex- and age-specific social, mood, and cognitive impairments in offspring.

**Methods:**

Timed pregnant Long-Evans rats were exposed to CIH or room air normoxia from GD 15-19. Behavioral testing of offspring occurred during either puberty or young adulthood. To examine ASD-associated phenotypes, we quantified ASD-associated behaviors (social function, repetitive behaviors, anxiety-like behaviors, and spatial memory and learning), hippocampal activity (glutamatergic NMDA receptors, dopamine transporter, monoamine oxidase-A, EGR-1, and doublecortin), and circulating hormones in offspring.

**Results:**

Late gestational CIH induced sex- and age-specific differences in social, repetitive and memory functions in offspring. These effects were mostly transient and present during puberty. In female pubertal offspring, CIH impaired social function, increased repetitive behaviors, and increased circulating corticosterone levels, but did not impact memory. In contrast, CIH transiently induced spatial memory dysfunction in pubertal male offspring but did not impact social or repetitive functions. Long-term effects of gestational CIH were only observed in female offspring, wherein CIH induced social disengagement and suppression of circulating corticosterone levels in young adulthood. No effects of gestational CIH were observed on anxiety-like behaviors, hippocampal activity, circulating testosterone levels, or circulating estradiol levels, regardless of sex or age of offspring.

**Conclusions:**

Our results indicate that hypoxia-associated pregnancy complications during late gestation can increase the risk for ASD-associated behavioral and physiological outcomes, such as pubertal social dysfunction, corticosterone dysregulation, and memory impairments.

## Plain Language Summary

Sleep apnea during late pregnancy is a common pregnancy complication associated with autism spectrum disorders (ASD) in children. ASD associated behaviors associated include decreased social skills, memory issues, anxiety, and compulsivity. It is unknown if there is a cause and effect relationship between sleep apnea during late pregnancy and behavioral changes in offspring, such as impaired social function, memory dysfunction, anxiety, and compulsivity. In this study, we exposed pregnant rats to a model of sleep apnea called chronic intermittent hypoxia during the last five days of pregnancy, and then examined the behavior of the offspring during puberty and young adulthood. In this study we found that sleep apnea during late pregnancy had long-term effects in the offspring. Notably, we only observed long-term effects of sleep apnea during late pregnancy in female offspring but not male offspring, in which social impairments persisted into young adulthood. Social impairments in girls are more difficult to clinically diagnose than social impairments in boys, which may be related to the underdiagnosis of ASD in females.

## Background

Hypoxia is a main characteristic of gestational sleep apnea and a culprit for other obstetric complications, such as gestational diabetes and preeclampsia [1–4]. Gestational sleep apnea affects 8–26% of pregnancies [5–8], and is associated with impaired fetal development underlying fetal central nervous system (CNS) dysfunction, growth restriction, and organ damage [9–13]. In addition to adverse perinatal outcomes, gestational hypoxia can increase the risk for chronic disorders, such as neurodevelopmental disorders in offspring later in life [14]. Specifically, offspring exposed to hypoxia *in utero* have a 35% increased risk of developing an autism spectrum disorder (ASD) [15–18]. ASD is defined by persistent impairments in social function with restricted or repetitive patterns in behavior [19]. ASD presents during early childhood with prevalence rates in the United States at approximately 1 in 44 children [20].

Boys are 3–4 times more likely to be diagnosed with ASD than girls [20, 21], though this sex difference may reflect distinct symptoms or differential timing of symptom presentation in girls as compared to boys [22, 23]. In girls, the onset of ASD symptoms has been observed during late puberty, although many remain undiagnosed until adulthood [22, 23]. Puberty is a critical period for social, endocrine, and cognitive maturation [24, 25]. Puberty has been suggested as a period of increased vulnerability to social and psychological stressors in individuals with ASD [26]. Further, precocious puberty has been associated with ASD in girls [21]. Additionally, it is more difficult to diagnose social impairments in girls due to the increased ability to camouflage (i.e., perform socially ‘appropriate’ behaviors) [28, 29] and enhanced verbal function compared to boys with ASD [30]. This camouflaging (e.g., masking) ability can persist into adulthood [31], increasing the likelihood of women living with ASD to not receive appropriate diagnosis and supportive care. Sex also plays a key role in ASD comorbidities (e.g., anxiety and intellectual disability) [19, 20, 32]. Anxiety has been diagnosed in up to 82% of children with ASD [33], especially in girls [33, 34]. Intellectual disability has been diagnosed in up to 91% of children with ASD [33]. However, the role of sex in intellectual disability in ASD is unclear, as data for intellectual disability indicates increased prevalence in boys [35], while other studies have observed increased intellectual disability in girls [20].

These sex differences in ASD could be linked to the CNS [36], such as brain region specific-, neurotransmitter-, and steroid hormone dysregulation. The hippocampal brain regions are involved in learning, memory, and social function [37]. Hippocampal-mediated social and cognitive dysfunctions are associated with ASD [37, 38]. Hippocampal-mediated dysfunctions may be associated with changes in neurotransmitters and circulating hormones. Prior studies have found differences in glutamatergic, dopaminergic, and serotonergic pathways in ASD [39–43]. Differences in steroid hormones, (e.g., testosterone, cortisol, estrogen) during gestation and later in life may also contribute to the phenotype of ASD [44–48]. The intensity of social impairments, repetitive behaviors, and precocious puberty in ASD has been correlated to testosterone and estradiol levels [27, 49, 50], Further, elevated diurnal cortisol may contribute to comorbid anxiogenic conditions [51, 52].

Since gestational hypoxia can have a long-lasting impact on offspring physiology, brain function, and behavior, it is important to understand the influence of sex and age on gestational hypoxia-associated chronic outcomes. To examine these relationships, we used a chronic intermittent hypoxia (CIH) protocol during late gestation in rats to model late gestational sleep apnea [9]. We chose this model of gestational CIH as the incidence of gestational sleep apnea is higher during late gestation [6, 8], during which time fetal CNS development is occurring [53, 54]. We were the first to show that CIH from gestational days 15–19 induced sex-specific effects on offspring brain maturation in rats [9]. It is unknown how this relatively short CIH exposure during late gestation impacts social, mood, and cognitive function in the offspring, and if sex or age mediate these effects. Therefore, we hypothesized that late gestational CIH would induce sex- and age-specific cognitive impairments, steroid hormone dysregulation, and hippocampal dysfunction in offspring. We quantified the impact of late gestation CIH on offspring behaviors, steroid hormone levels, and hippocampal cellular function (e.g., neurotransmitters, neuronal activity, neurogenesis).

## Methods

### Animals

This study was part of a larger study that examined the impact of late-stage gestational hypoxia on maternal and offspring physiology [9]. All experiments were conducted using timed pregnant Long-Evans rats (aged 8–10 weeks, Charles River, Wilmington, MA). Dams arrived at animal facilities on gestational day (GD) 5–7, in which GD 0 was when the sperm plug was observed. Dams were single housed in a 12h:12h light/dark cycle with lights on at 0900 h and were provided food and water *ad libitum*. Dams were allowed to habituate for 4 days prior to the initiation of the chronic intermittent hypoxia (CIH) protocol that was performed during late gestation (GD 15–19) prior to delivery (GD 22–23). Maternal and fetal biometric analyses are reported in our prior publication [9]. Following delivery, the sex of the offspring was determined via anogenital distance and confirmed prior to weaning. Litters were reduced to 8 pups/litter and, when possible, to equal number of males and females. Weaning occurred on postnatal day (PND) 28. Approximately 4 male and 4 female pups in each litter were randomly assigned to either pubertal (PND 40–45) or young adult (PND 60–65) offspring experimental groups. Based on gestational exposure to hypoxia or normoxia (room air), offspring were included in the following treatment groups. Puberty: Male Normoxic (n = 12), Male CIH (n = 16), Female Normoxic (n = 10), Female CIH (n = 11); Young adult: Male Normoxic, (n = 13) Male CIH (n = 13), Female Normoxic (n = 10), Female CIH (n = 9).

Pubertal and young adult offspring were pair housed with a littermate of the same sex. Male and female offspring were housed in separate rooms in our animal facility. Offspring were housed in rooms on a 12-hour (h) reverse light cycle where lights were off at 0900 h. Reverse lighting allowed behavioral testing to be conducted during the offspring’s active phase of the circadian cycle. Food and water were provided *ad libitum* for all rats. To acclimatize the offspring to operator handling and reduce stress responses during behavior testing, offspring were handled daily, beginning approximately 10 days prior to the start of behavior testing. At the conclusion of behavior testing, the offspring were anesthetized with 2–3% isoflurane and euthanized via decapitation on PND 48 (puberty) or PND 66 (young adult). All offspring were euthanized during the active phase of the circadian cycle between 0900 h and 1100 h. All experiments were conducted in agreement with the Guide for the Care and Use of Laboratory Animals of the National Institutes of Health and the ARRIVE guidelines. These protocols were approved by the Institutional Animal Care and Use Committee of the University of North Texas Health Science Center.

### Chronic intermittent hypoxia protocol

Timed pregnant female rats were assigned to receive CIH (n = 8) or normoxic (NORM; n = 8) treatments for 8 h starting at 0900 h during their sleep phase of the circadian cycle on GD 15–19. Four days prior to the initiation of the CIH protocol, the home cages (clear plastic containers with sufficient bedding and nesting materials for the dams) of the timed pregnant females were placed into Oxycycler chambers (76.2x50.8x50.8cm, BioSpherix, Lacona, NY, USA) to acclimatize the dams to the chambers under normoxic (room air) conditions. The CIH protocol consists of oxygen reduction from 21% (room air) to 10% oxygen, then returned to 21% oxygen in 6-minute (min) cycles per hour (10 cycles/hour) over 8 hours/day for a period of 5 days, as previously described [9]. Following the end of the CIH protocol (GD 19), the cages containing the dams were left in the Oxycycler chambers with the chambers open to room air, and the dams were not disturbed until 24 hours following delivery for sexing of the pups.

### Behavioral Tasks

Behavioral studies were conducted either during puberty (PND 40–45) or young adulthood (PND 60–65) over the course of one week during the active phase of their circadian cycles, from 0945 h to 1700 h. This cross-sectional design, in which rats were only tested either during puberty or young adulthood, was performed to prevent learning due to prior experience with these tasks (i.e., test battery effects). Notably, test battery effects have been reported in open field tests [71–73] and Morris water maze [71, 72], which are behavior tests used in this study. Therefore, to avoid this confound, one behavioral task per index of interest was performed and rats were assessed at either puberty or young adulthood. The order of the behavior tests was randomized. Male offspring were tested at least one hour before female offspring to avoid potential confounding effects of pheromones on behavior. All testing equipment (e.g., marbles, arenas, tanks) were thoroughly cleaned with 70% ethanol between each rat. All behavior studies were conducted under red lighting and recorded for later analysis by an investigator blinded to treatment groups. Behavior tests were used to assess ASD-associated behaviors, such as repetitive behaviors (marble burying test), social function (social behaviors, social disengagement, social withdrawal, aggression), anxiety-like behaviors (center entries and center duration in an open field, exploration), and spatial learning and memory (Morris water maze).

### Marble burying test

This test was used to observe repetitive behaviors [74, 75]. To conduct this test, the floor of the testing arena (50x25x30 cm) was thoroughly covered with 1 cm of rodent bedding litter to allow the rats to easily bury marbles. Twenty marbles of similar color and size (1.5 cm) were positioned in a 4x5 grid spaced evenly on one side of the arena base, and a pre-test photograph was taken. At the beginning of the test, a single rat was placed on the opposite side of the arena facing away from the marbles (Supplemental Fig. 1A). Each rat was given 10 minutes to explore and interact with the marbles. Operators visually monitored the experiment and manually recorded behaviors. After 10 minutes, the rat was removed and the arena was photographed for later quantification of the number of buried marbles. A marble was considered buried if 75% or more of the marble was covered with bedding litter. Post hoc quantification was performed by an experimenter blinded to the treatment of the rats by examining the photographs taken before and after the test.

### Social function

To quantify social behaviors, social disengagement, and social withdrawal in a group social setting, rats were placed into a novel arena (50x25x30 cm) with novel conspecific rats of the same sex for 10 minutes. Due to the uneven numbers in some of the study groups and to maintain the novelty of the arena, social group settings were either 2 or 3 rats of the same sex. We observed no differences in behaviors due to these social group numbers (Supplemental Table 1). The arena was lined with litter bedding (1.5 cm). To allow the rats to retreat from the novel arena, a foil covered semi-cylindrical tube (6.5x9x20.5 cm) was placed into the arena, which was large enough for rats to enter and hide. Behaviors assessed included: 1) social behaviors (sniffing + following + climbing_under_+climbing_over_ = social behavior composite score) [76, 77], 2) social disengagement behavior (percentage of nose-to-nose engagements where the rat was the first to disengage from interaction with a conspecific; Supplemental Fig. 1B), 3) social withdrawal (rats that did not perform nose-to-nose interactions with conspecifics), 4) exploratory behaviors (rearing + digging + tube_climb_+tube_sniff_+tube_chew_) [78, 79], 5) aggressive behaviors (kicking + biting + dominance posture + boxing + fighting = aggressive behavior composite score) [80–83]. Due to technical issues with video recordings during the social behavior testing, footage for 9 young adult females (normoxic n = 4; CIH n = 5) was lost and not available for analysis for social disengagement behavior, social withdrawal, and exploratory behavior.

### Open field

Anxiety-like behaviors were assessed using a novel open field arena (40.64x40.64x38.1 cm) with the bidirectional main field bar (San Diego Instruments Photobeam Activity System (PAS)-Open field arena). Rats were allowed 10 minutes in the novel open field arena to explore. Anxiety-like behaviors can be assessed by the time spent (duration) or entries into the center of the open field [84]. To quantify these behaviors, a center zone was created within the PAS system to denote the number of entries (frequency) to the zone and time spent (duration) within the zone versus time spent around the edge [85].

### Morris water maze

To examine spatial memory and learning, the Morris water maze test was used according to our published protocols [85]. On the first day of the Morris water maze, the rats were trained to swim in a pool filled with opaque water (23-25°C) to a visible platform approximately 1 cm above the surface of the water and remain on the platform for 20 seconds until removed by the operator. The following three days (day 2–4 of testing) consisted of 3 trials/day with a 10 min inter-trial interval per rat to train the rats to locate a submerged target platform (learning phase). For each trial, a rat was placed into the pool at a randomly assigned point, equidistant from the target. Rats were allowed 90 seconds to locate the target with a trial ending when either the rat located the target and climbed onto it, or 90 seconds had passed. Once the target was located, each rat was allowed to sit on the platform and observe visual cues placed on the walls to aid in the formation of spatial memory for 20 seconds. After the 20 seconds passed, the rat was removed from the water maze and placed into a carrier to dry and wait for the next trial. Rats that did not locate the hidden target were guided to the target by means of the operator tapping on the target until the rat swam to and climbed onto the platform. Twenty seconds were then provided for the rat to observe spatial cues and rest, and then the rat was returned to the carrier. The target remained in the same location throughout all 3 days of training. On day 5, each rat was administered a probe trial to test for spatial memory retention. During the probe trial, the underwater platform was removed. Each rat was placed into the water at one of the pre-determined random entry points and allowed 30 seconds to swim to the target location and search for the platform. At the end of 30 seconds, the platform was returned to its original location and the rat was allowed 20 seconds to sit on the platform to reduce stress. Latency and pathlength to the target were recorded using ANY-maze software (v. 5.14, Stoelting Co.). Latency and pathlength to the target during the probe trial were used as indicators of spatial memory retention, and latency on each day leading up to the probe trial was used to measure learning.

### Sample collection

To collect tissue and blood samples, rats were anesthetized with isoflurane (2–3%) and decapitated during the first 2 hours of the rats’ active phase of the circadian rhythm [9, 85, 86]. Trunk blood was collected in EDTA tubes, and then centrifuged at 2,000 x g for 10 minutes at 4°C to collect plasma. Plasma was stored at −80°C. Each brain was quickly removed, flash frozen in 2-methlylbutane, and sliced into 1-mm coronal sections using a brain matrix (RBM-4000C, ASI Instruments, Warren, MI). Using blunt 20-guage needles attached to 1 mL syringes for brain region microdissection [9, 85–88], the dentate gyrus (DG) and CA1 region of the dorsal hippocampus (− 5.30 mm from Bregma) were isolated according to Paxinos and Watson’s brain atlas [89]. Micro-dissected brain samples were placed into microcentrifuge tubes to be stored at −80°C until protein analysis.

### Hormone assays

Steroid hormones were extracted from plasma via acetonitrile preparation [90]. Briefly, plasma samples were diluted 2:3 in HPLC grade acetonitrile, then vortexed at room temperature. After 10 minutes, the plasma samples were centrifuged at 17,000 × *g* at 4°C for 5 minutes. Supernatants were collected and dried by vacuum centrifugation. All samples were reconstituted in the appropriate assay buffer prior to use in ELISA assay according to manufacturer’s instructions. Commercially available, competitive immunoassay ELISA kits were used for quantitative determination of circulating testosterone (ADI-900-065, Enzo Life Sciences Inc., Farmingdale, NY), corticosterone (ADI-900-097, Enzo Life Sciences Inc., Farmingdale, NY), and estradiol (RTC009R, BioVendor, Czech Republic) according to manufacturer’s instructions [91, 92]. The intra-assay coefficient of variation for testosterone ELISA was 7.8% with an inter-assay coefficient of variation at 9.3%. The sensitivity of this testosterone ELISA was 5.67 pg/ml at the 2-standard deviation (s.d.) confidence limit. The intra-assay coefficient of variation for corticosterone ELISA was 6.6% with an inter-assay coefficient of variation at 7.8%. The sensitivity of the corticosterone ELISA was 26.99 pg/ml at the 2 s.d. confidence limit. The intra-assay coefficient of variation for estradiol ELISA was 6.1% with an inter-assay coefficient of variation at 7.0%. The sensitivity of estradiol ELISA was 2.5 pg/ml at the 2 s.d. confidence limit. Reported values are mean ± s.d.

### Tissue sample preparation, electrophoresis, and western blots

For protein analysis, frozen tissue samples were thawed in RIPA buffer (VWR, cat #N653) containing (per 0.5 ml): 2.5 μl Halt^™^ protease and phosphatase inhibitor (Thermo Scientific, cat #78442), 1 μl 0.5 M ethylenediaminetetraacetic acid (EDTA, Sigma-Aldrich), and 1 μl 0.5 mM dithiothreitol (DTT, Sigma-Aldrich) for homogenization, as previously described [9, 88, 93–95]. Total protein concentration levels in the homogenate were determined using Pierce BCA Protein Assay (Thermo Fisher, cat #23225). Samples were denatured with β-mercaptoethanol and boiled at 100°C for 5 min. Equal volumes of denatured tissue samples containing 30 μg protein were loaded into a Bio-Rad 4–15% polyacrylamide gel. The gel then underwent electrophoresis at 25 milliamps (mA) in a tris-glycine running buffer followed by overnight transfer at 4°C onto a PVDF membrane at 50 mA. Following 30 minutes washing, membranes were blocked for 30 minutes with 5% nonfat milk in tris-based saline (TBS)-Tween (TBST) at room temperature. Membranes were then transferred to 1% nonfat milk TBST solutions containing specific primary antibodies and incubated overnight at 4°C. Primary antibodies used on CA1 hippocampal micro-punched tissue included DAT-HRP (Santa Cruz, SC-32259 1:5000), MAO-A (Abcam, ab126751 1:1000), NR2A (Antibodiesinc.com, 73–303 1:500). For DG tissue samples, primary antibodies for doublecortin (Santa Cruz, sc-271390 1:500) and Egr-1 (Santa Cruz, sc-515830 1:500) were used. Afterwards, membranes were washed in 10-minute increments for 30 minutes, and then incubated in 1% milk TBST-secondary antibody solutions including HRP-conjugated horse anti-mouse (Cell Signaling, 7076P2 1:2500) or HRP-conjugated goat anti-rabbit (Cell Signaling, 7074P2 1:5000) at room temperature for 1 hour. For protein normalization, we used beta-actin primary antibody (GeneTex, GTX 629630 1:3000), which was incubated for 1 hour at room temperature. Protein bands were visualized using West Pico (Thermoscientific, cat #34580) or West Fempto (Thermoscientific, cat #34095) enhanced chemiluminescence detection assay in a Syngene G:Box system using FlourChem HD2 AIC software as previously described [9]. NIH Image J software (version 1.48v) was used to quantify band densitometry of protein of interest normalized to beta actin band densitometry. The following proteins of interest were quantified: 1) glutamatergic NMDA receptors (NR2A) that mediate excitatory neuronal synaptic activity and are associated with spatial memory and social behaviors [55, 56]; 2) dopamine transporter (DAT), a monoamine neurotransmitter transporter [57] that can impact memory function, anxiety, and social function [58–60]; 3) monoamine oxidase-A (MAO-A), an enzyme that catalyzes monoamines (e.g., serotonin, norepinephrine, and dopamine) and is associated with memory and social function [60–62]; 4) early growth response protein 1 (EGR-1), a transcription factor expressed in numerous cell types that acts to mediate cellular activity [63, 64], with hippocampal expression associated with memory, anxiety and neuropsychiatric disorders [65, 66]; and 5) doublecortin, a neurogenesis marker associated with memory, anxiety and social function [67–70].

### Statistical analysis

Significance was defined as p ≤ 0.05. Statistical analyses were conducted in IBM SPSS (SPSS v. 29.0.0, IBM, 2022). Data distribution was tested using the Shapiro-Wilk test. Data with non-Gaussian distribution were normalized by square root transformation through the SPSS transformation function (x = sqrt(x)). Outliers greater than 2 s.d. from the mean were removed from analysis. Since social withdrawal was exhibited by a low number of animals, Fisher’s exact test was used to determine the impact of gestational CIH on social withdrawal [96]. For all other behavior tests, ELISAs, and protein analyses, the main effects and interactions were examined using two-way ANOVAs with the factors of treatment and sex within either the puberty or young adult groups, wherein we provide the F values, degrees of freedom, p-values, and η^2^ (measure of effect size). To examine the effect of age, two-way ANOVAs with the factors of treatment and age within either male or female groups were performed. Following ANOVA, a Fisher’s LSD posthoc test was used to determine specific group differences. Results are presented as mean ± S.E.M. unless otherwise indicated.

## Results

### Late gestational CIH induced social impairments in female offspring without affecting aggressive or exploratory behaviors

**Figure 1:**
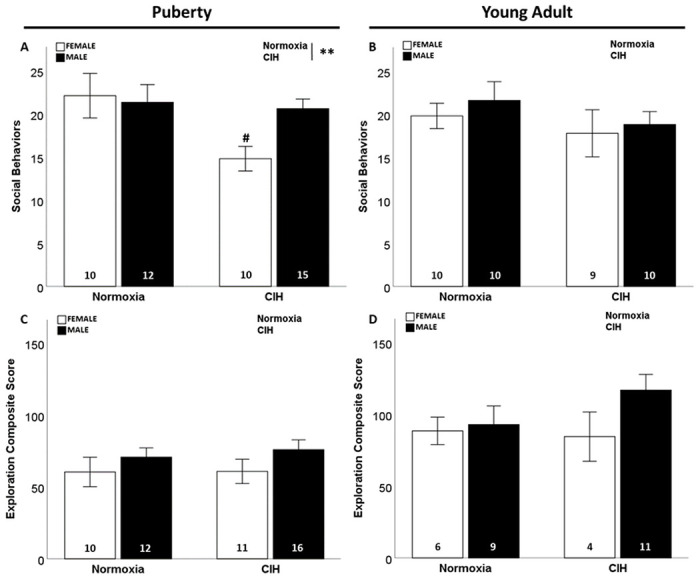
Social and exploratory behaviors. Gestational CIH decreased social behaviors in female rats during puberty (A) but not during young adulthood (B). No effect of gestational CIH was observed on male rats during puberty (A) or young adulthood (B). No effect of sex was observed on social behaviors (A, B). No effect of gestational CIH or sex was observed in exploratory behavior (C, D). Analyzed by Two-way ANOVA with Fisher’s LSD multiple comparisons tests. ANOVA significance indicated by: ** = CIH; Post-hoc significance indicated by: # versus normoxic female; p≤0.05

We observed that gestational CIH significantly reduced social behaviors displayed by pubertal rats (F_1, 43_=4.934; p = 0.032; η^2^ = 0.093; Fig. 1A), especially in pubertal females (p ≤ 0.05). However, this effect of gestational CIH on social behaviors was not maintained in young adult rats (Fig. 1B). No effects of sex or age were observed on social behaviors.

We observed no effects of gestational CIH or sex on exploratory behavior in pubertal or young adult rats (Fig. 1C–D). We found increased exploratory behavior by young adult females compared to pubertal females (F_1, 27_=5.001; p = 0.034; η^2^ = 0.156; Supplemental Fig. 2A). This effect was also observed in young adult males compared to pubertal males (F_1, 44_=11.943; p = 0.001; η^2^ = 0.200; Supplemental Fig. 2B).

**Figure 2:**
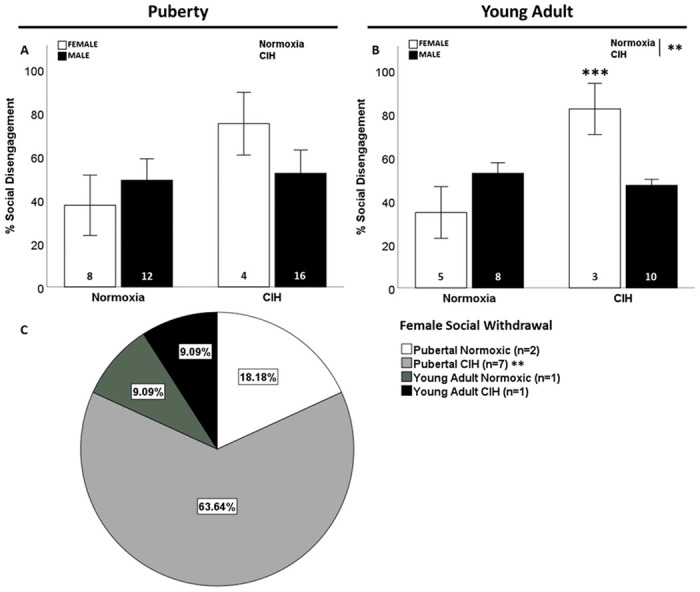
Social disengagement. No effect of gestational CIH or sex was observed on social disengagement in pubertal rats (A). Gestational CIH only increased percentage of young adult female rats that exhibited social disengagement (B). Social withdrawal was significantly associated with gestational CIH only in pubertal females (C). Social disengagement analyzed by Two-way ANOVA with Fisher’s LSD multiple comparisons tests (A, B). Relationship between social engagement and CIH measured by Fisher’s exact test (C). Significance indicated by: ** = CIH, *** = interaction; p≤0.05

No effects of gestational CIH or sex on social disengagement were observed in pubertal rats (Fig. 2A). In contrast, there was a significant interaction between gestational CIH and sex on social disengagement in young adult rats (F_1, 26_=14.472; p < 0.001; η^2^ = 0.307; Fig. 2B), in which gestational CIH increased social disengagement in young adult females. Further, late gestational CIH increased social disengagement in pubertal and young adult females (F_1, 20_=7.540; p = 0.014; η^2^ = 0.319; Supplemental Fig. 3A). No effect of CIH or age was observed in male offspring (Supplemental Fig. 3B). Late gestational CIH increased social withdrawal in pubertal females (p ≤ 0.05; Fig. 2C). No males displayed social withdrawal.

Since developmental dysfunction and altered steroid hormones can impact aggression in pubertal and young adult rats [80–83], aggressive behaviors were recorded. Aggressive behaviors include kicking, biting, dominance posture, boxing and fighting [80–82]. Offspring displayed little to no aggressive behaviors, regardless of gestational CIH, sex, or age (Supplemental Table 2).

### Late gestational CIH increased repetitive behaviors only in pubertal females

**Figure 3:**
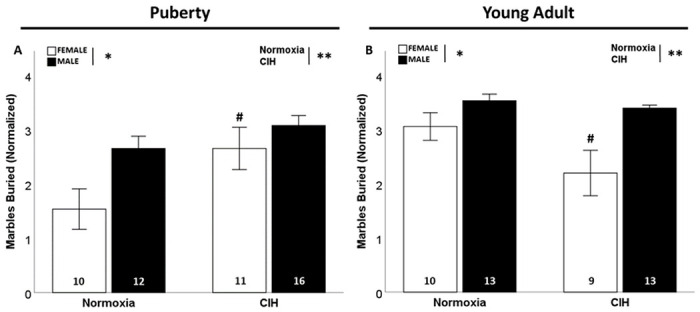
Repetitive behaviors. Gestational CIH increased marble burying in pubertal females (A) and young adult females (B). Males buried more marbles than females, regardless of age (A, B). Normalized by square-root transformation (A, B). Analyzed by Two-way ANOVA with Fisher’s LSD multiple comparisons tests. ANOVA significance indicated by: * = sex, ** = CIH; Post-hoc significance indicated by: # versus normoxic female, p≤0.05

In pubertal rats, there was a significant effect of gestational CIH on marble burying (F_1, 49_=7.215; p = 0.010; η^2^ = 0.118; Fig. 3A), where gestational CIH exposed pubertal females buried more marbles than normoxic pubertal females (p ≤ 0.05). There was also a significant effect of sex on marble burying (F_1, 49_=7.205; p = 0.010; η^2^ = 0.118; Fig. 3A) in pubertal rats, where males buried more marbles than females. Conversely, in young adulthood the significant effect of CIH (F_1. 45_=5.204; p = 0.028; η^2^ = 0.082; Fig. 3B) is reversed, with fewer marbles buried by offspring exposed to gestational CIH, specifically by females (p ≤ 0.05). Similar to puberty, males during young adulthood (F_1, 45_=14.761; p< 0.001; η^2^ = 0.232; Fig. 3B) buried more marbles than females. There was a significant interaction between gestational CIH and age in females (F_1, 36_=7.322; p = 0.010; η^2^ = 0.161; Supplemental Fig. 4A), in which gestational CIH increased repetitive behaviors only in pubertal females. Age also affected marble burying in males, as young adult males buried more marbles than pubertal males (F_1, 50_=13.669; p < 0.001; η^2^ = 0.202; Supplemental Fig. 4B).

### Late gestational CIH did not induce anxiety-like behaviors

No effect of gestational CIH was observed on center duration in pubertal offspring (Fig. 4A) or young adult offspring (Fig. 4B). Pubertal males spent more time in the center of the open field arena than females during puberty (F_1, 45_=8.759; p = 0.005; η^2^ = 0.153; Fig. 4A), in which normoxic males spending more time than normoxic or gestational CIH exposed females (p ≤ 0.05). Young adult males spent more time in the center than females (F_1, 41_=5.736; p = 0.021; η^2^ = 0.113; Fig. 4B), with gestational CIH exposed males spending more time in the center than normoxic or gestational CIH exposed females (p ≤ 0.05). There was a significant effect of age on time spent in the center of the open field, where young adult females (F_1, 36_=29.056; p < 0.001; η^2^ = 0.436; Supplemental Fig. 5A) and males (F_1, 50_=25.264; p < 0.001; η^2^ = 0.311; Supplemental Fig. 5B) spent more time than pubertal rats. Gestational CIH exposed young adult males spent more time in the center than either normoxic males or gestational CIH exposed pubertal males (p ≤ 0.05). We observed no effects of gestational CIH or sex on the number of center entries displayed by offspring (Fig. 4C, D). Similar to center duration, we observed that aging is associated with increased center entries, as shown by more entries performed by young adult females (F_1, 36_=42.528; p < 0.001; η^2^ = 0.531; Supplemental Fig. 5C) and young adult males (F_q 50_=17.729; p < 0.001; η^2^ = 0.255; Supplemental Fig. 5D) compared to pubertal rats.

### Late gestational CIH only impacted spatial memory function in pubertal male rats

**Figure 5:**
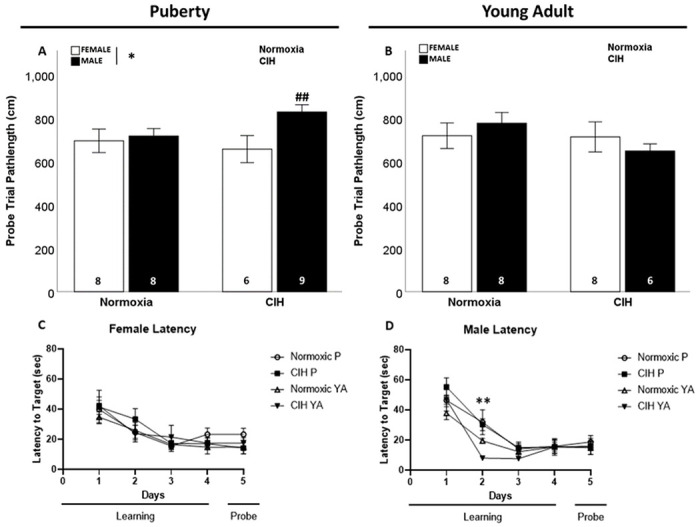
Spatial learning and memory. Pubertal males had longer pathlength to target during Morris water maze probe trial (A), with the sex difference observed primarily in gestational CIH exposed pubertal males. No effect of sex or gestational CIH was observed on pathlength in young adult rats (B). No effect of gestational CIH on latency to target was observed in females regardless of age (C). Age improved latency to target during day 2 of learning in males with no effect of age observed on other days (D). Analyzed by Two-way ANOVA with Fisher’s LSD multiple comparisons tests. ANOVA significance indicated by: * = sex; ** = age; Post-hoc significance indicated by: ## = versus CIH female; p≤0.05; *P = Puberty, YA = Young Adult*

We observed sex differences in pathlength to the target, in which pubertal females exhibited shorter pathlength to the target than pubertal males (F_1, 27_=4.495; p = 0.043; η^2^ = 0.129; Fig. 5A), specifically pubertal females exposed to gestational CIH had shorter pathlengths than pubertal males exposed to gestational CIH (p ≤ 0.05). However, gestational CIH did not affect pathlength to the target in pubertal (Fig. 5A) or young adult offspring (Fig. 5B). No sex differences in spatial memory were observed in young adult rats. No effect of age on spatial memory was observed in females (Supplemental Fig. 6A). In male offspring, the effects of gestational CIH on spatial memory were dependent on age. Specifically, gestational CIH increased pathlength to the target in pubertal males, but decreased pathlength in young adult males (F_1, 27_=9.375; p = 0.005; η^2^ = 0.242; Supplemental Fig. 6B). Although we observed significant effects of gestational CIH, sex, and age on spatial memory-associated pathlength to target, we did not observe any effects on latency to the target during the probe trial (Supplemental Table 3).

There were no sex differences or effects of gestational CIH on learning (latency to target) in offspring (Fig. 5C, D). However, we did observe faster learning (shorter pathlengths to target) in young adult males on day 2 of training compared to pubertal males (F_1, 27_=12.109; p = 0.002; η^2^ = 0.290; Fig. 5D) or young adult females (F_1, 26_=6.824; p = 0.015; η^2^ = 0.187), though this was not observed on any other days of testing. This effect of age on learning (i.e., shorter pathlengths to target) was not observed in female offspring (Fig. 5C).

#### Late gestational CIH did not impact expressions of proteins associated with social and cognitive function within the dorsal hippocampus

Of the proteins that we examined, gestational CIH did not impact protein expression (Table 1; Supplemental Fig. 7), indicating that gestational CIH exposure from GD 15–19 does not impact cellular function in the dorsal hippocampus of offspring. Although we did not observe any effects of late gestational CIH on dorsal hippocampal cellular activity, there were sex– and age-dependent effects on DAT, NMDA receptors, and EGR-1 protein expression (Table 1). Sex only affected DAT expression in the CA1, where young adult males had significantly higher expression compared to young adult females (F_1, 20_=8.122, p = 0.010, η^2^ = 0.277). We also observed age differences: 1) decreased DAT (F_1, 20_=4.484, p = 0.047, η^2^ = 0.179) and decreased NR2A (F_1, 20_=6.716, p = 0.017, η^2^ = 0.251) in the CA1 of young adult females compared to pubertal females, 2) decreased EGR-1 (F_1, 20_=11.219, p = 0.003, η^2^ = 0.358) in the dentate gyrus of young adult females compared to pubertal females, and 3) decreased EGR-1 (F_1, 20_=36.500, p < 0.001, η^2^ = 0.627) in the dentate gyrus of young adult males compared to pubertal males.

### Late gestational CIH differentially affected circulating corticosterone levels in females

We observed sex differences in circulating testosterone and corticosterone levels, as well as an effect of gestational CIH on corticosterone levels (Table 2). No effect of gestational CIH, sex, or age was observed on plasma estradiol levels (Table 2). No effect of gestational CIH was observed on plasma testosterone levels. However, we observed expected sex differences in testosterone levels: 1) higher testosterone levels in pubertal males compared to pubertal females (F_1, 32_=30.166; p < 0.001; η^2^ = 0.480) and 2) higher testosterone levels in young adult males compared to young adult females (F_1, 35_=41.037; p < 0.001; η^2^ = 0.526). Testosterone levels also increased with age in females (F_1, 31_=10.739; p = 0.003; η^2^ = 0.235) and males (F_1, 36_=31.029; p < 0.001; η^2^ = 0.462).

To examine the impact of gestational CIH on circulating hormones associated with the stress response, we quantified plasma corticosterone levels (Table 2). Gestational CIH increased circulating corticosterone in pubertal rats (F_1, 31_=5.407; p = 0.027; η^2^ = 0.094), particularly in female offspring (p ≤ 0.05). A sex difference was also observed in pubertal rats, with higher plasma concentrations of corticosterone observed in female offspring than male offspring (F_1, 31_=19.247; p < 0.001; η^2^ = 0.338). In young adulthood, the effect of gestational CIH on circulating corticosterone levels was reversed, with lower corticosterone observed (F_1, 35_=5.313; p = 0.027; η^2^ = 0.061), specifically in young adult females (p ≤ 0.05). The sex difference in corticosterone levels was maintained into young adulthood, with higher levels of corticosterone observed in young adult female offspring than young adult male offspring (F_1, 35_=43.840; p < 0.001; η^2^ = 0.501). In female offspring, an interaction between age and gestational CIH was observed, with plasma corticosterone levels increasing in gestational CIH exposed pubertal offspring but decreasing in gestational CIH exposed young adult offspring (F_1, 33_=8.343; p = 0.007; η^2^ = 0.172). No effect of age on corticosterone levels was observed in male offspring.

## Discussion

Late gestational CIH induced sex- and age-specific differences on social and cognitive function in offspring, in which social dysfunctions were observed in female offspring while cognitive dysfunction was observed in male offspring. Late gestational CIH effects on social and cognitive behaviors were mostly transient and present only during puberty, except in females that showed sustained social disengagement and suppressed corticosterone levels during young adulthood. In pubertal female offspring, late gestational CIH transiently impacted social function associated behaviors: 1) impaired social behaviors (sniffing, following, climbing over/under another rat); 2) increased social withdrawal (absence of nose-to-nose interactions with conspecifics); and 3) increased repetitive behaviors (marble burying). In addition, circulating corticosterone levels were increased by late gestational CIH in pubertal female offspring. In contrast, late gestational CIH in pubertal male offspring transiently induced cognitive dysfunction (increased pathlength). We also observed no effects of late gestational CIH on anxiolytic, aggressive, or exploratory behaviors. Long-term effects of late gestational CIH were only observed in females, in which increased social disengagement, decreased marble burying, and decreased circulating corticosterone levels were observed in young adulthood. Interestingly, the effects on marble burying and corticosterone in young adulthood were opposite to the effects observed in puberty, while increased social disengagement was sustained.

The current study is the first to examine the effects of late gestational CIH on social function, anxiety, and cognitive function in rat offspring. Further, this is the only study to use Long-Evans rats, as prior studies used either Sprague Dawley or Wistar rats (Supplemental Table 4). We chose to use Long-Evans rats in our studies, as this rat strain exhibits increased behavior (activity, cognitive function, exploration), greater stress reactivity, and increased sensitivity to hypoxia compared to other rat strains [92, 97, 98]. Additionally, Long-Evans rats are commonly used to study prenatal brain development [99–101]. Brain development during pregnancy occurs in three stages (Supplemental Table 4): 1) stage 1 (GD 1–10) in which the neural tube is formed and is comparable to the first three weeks of human gestation [102, 103]; 2) stage 2 (GD 10–15) in which the establishment of cortical and subcortical brain regions occurs and is comparable to the first two months of human gestation [104–106]; and 3) stage 3 (GD 15–22) in which cortical and subcortical brain maturation occurs and is comparable to the last 7–8 months of human gestation [103, 106, 107]. We have previously shown that short-term, late gestational CIH (GD 15–19) had sex- and age-specific effects on nigrostriatal pathway maturation in pubertal female and young adult male offspring [9]. Impairment of the nigrostriatal pathway is associated with multiple neuropsychiatric disorders, such as cognitive dysfunction [108]. ASD [39, 108], and mood disorders [39, 108]. Therefore, here we examined the impact of late gestational CIH on social function, anxiety, and cognitive function in offspring.

Prior studies investigating the impact of gestational hypoxia in rodent offspring (Supplemental Table 4) have found equivocal findings that range from social impairments in male offspring with no effects in female offspring [109, 110] to social impairments in female offspring with no effects in male offspring [76, 111] or social impairments in both sexes [112]. Reports of sex differences in cognition due to gestational hypoxia have been observed, with impairment observed in male offspring [109, 113], female offspring [114], or neither sex [76, 112, 115]. These behavioral differences in offspring exposed to gestational hypoxia may be due to the timing or the type of hypoxic insult, as they ranged in hypoxia exposure duration (1–21 days), hypoxia cycles (intermittent, sustained hypoxia), and intensity of lowered oxygen concentration (13%-5%). Our study utilized CIH (10 hypoxia cycles/hour/for 8 hours each day at 10% O_2_) during late gestation (GD 15–19). Although no other study has used gestational intermittent hypoxia exposure targeting brain development stage 3, prior studies using a sustained hypoxia protocol during this time have been conducted (Supplemental Table 4). Specifically, sustained hypoxia during this time period (GD 15–22) impaired social behaviors displayed by young adult female offspring and not young adult male offspring [76, 111], had no impact on anxiety-like behaviors in young adult male and female offspring [76, 116], and decreased repetitive behaviors in young adult female offspring [111]. However, there were differences between these prior studies and our current study, in which sustained hypoxia impaired cognitive function in young adult female offspring [114], decreased repetitive behaviors in young adult male offspring [76, 111], and increased anxiety-like behaviors in young adult male offspring [76, 111], indicating that the type of hypoxic exposure during this critical period of brain development is important.

The current study is the first to examine the impact of sex as a biological variable in behavior displayed by pubertal offspring exposed to late gestation hypoxic stress. A recent study examined the effects of acute gestational hypoxia (sustained, 6 hours) on GD 17 on pubertal male and female Sprague Dawley rat offspring, in which impaired cognitive function, impaired social function, and increased repetitive behaviors were observed [112]. However, the role of sex is unknown as sex differences were not assessed in this study [112]. These studies highlight that gestational brain development stage 3 is a vulnerable period important for social and cognitive function in both pubertal and young adult offspring.

Gestational hypoxia increases the risk for ASD by 35% [15–18]. Female offspring exposed to late gestational hypoxia exhibited increased social dysfunction and repetitive behaviors, which are associated with ASD [19, 33, 35]. We did observe sex differences in the display of ASD-associated behaviors, in which increased social dysfunction and repetitive behaviors were observed only in female offspring. Recently, reports have shown that girls are underdiagnosed and/or diagnosed with ASD later in life compared to boys [22, 23]. One of the reasons for underdiagnosis of ASD in females could be that the diagnostic tools are based on ASD presentation by boys [22, 117], but the behavioral presentations of ASD are different for boys and girls [19, 118]. Similar to our findings showing increased social dysfunction in female offspring, social dysfunction is present in girls diagnosed with ASD [28, 29, 118]. However, it is difficult to diagnose social dysfunction in girls with ASD due to their increased ability to camouflage or display socially ‘appropriate’ behaviors compared to boys with ASD [28, 29, 31]. Therefore, the current ASD prevalence rates of 1 in 44 children in the USA [20] may be higher due to the underdiagnosis in girls, as well as the 24.4% annual increase in prevalence rates for gestational sleep apnea hypoxia [119].

In addition to behavioral effects of late gestational hypoxia, we examined glutamatergic (NR2A), dopaminergic (DAT), and serotonergic (MAO-A) associated proteins in the CA1 region of the dorsal hippocampus, as these have been associated with social and cognitive impairments in ASD [39–43]. Similar to our prior publication that found no effects of late gestational CIH on protein expression within the substantia nigra (calpain enzymatic activity, caspase-3 enzymatic activity, tyrosine hydroxylase) [9], we observed no gestational CIH-associated changes in proteins associated with glutamatergic, dopaminergic, and serotonergic functions, regardless of sex or age of the offspring. We also examined markers for cellular activity (EGR-1) and neurogenesis (doublecortin) within the dentate gyrus region of the dorsal hippocampus, as cellular activity and neurogenesis within the dentate gyrus is important in mediating spatial memory [120, 121] and social function [67–70], as well as being associated with ASD [122, 123]. We found no effects of late gestational CIH on EGR-1 or doublecortin protein expression in the dentate gyrus, regardless of sex or age. Future studies need to be conducted to examine the electrophysiological properties (long-term potentiation and long-term depression) of dorsal hippocampal neurons and neurogenesis (bromodeoxyuridine, BrdU), as dysfunction in these cellular properties have been linked with ASD [122, 123].

ASD has been associated with altered circulating steroid hormones levels (e.g., testosterone, cortisol, estradiol) [27, 44–52]. We did not observe any effects on circulating testosterone levels or testosterone-associated behaviors (e.g., aggression) in offspring exposed to gestational CIH. Although increased testosterone levels in children with ASD has been associated with social dysfunction and aggressive behavior [44, 46, 51], other reports found no association between testosterone and pubertal ASD behaviors [47,124–126], Recently, the focus has been shifting to examining associations between ASD behaviors and other sex hormones, such as estrogens and cortisol [47, 127]. We found no effect of gestational CIH on circulating estradiol levels. We did find that gestational CIH impacted corticosterone levels. The association between cortisol and ASD is equivocal, in which some studies show an association between elevated cortisol associated with ASD [52, 126, 132] and other studies do not [47, 133]. Our findings that gestational CIH increased circulating corticosterone levels in pubertal females, coincides with findings that increased corticosterone levels have been observed in ASD-associated behaviors [52, 126, 132], such as social impairment and repetitive behavior. The decreased corticosterone levels observed in the young adult females indicates that the effect of gestational CIH on corticosterone may be transient, though social impairments remain.

We also observed several sex- and age-associated developmental effects on brain and behavior. Gestational CIH exposed pubertal female offspring had shorter pathlengths in the Morris water maze than gestational CIH exposed pubertal male offspring, an effect which was not sustained into young adulthood. Other groups using adult rats found no sex differences in Morris water maze performance [134–137], and found that females may have shorter pathlength and latency depending on experimental conditions like training duration and room lighting [137]. Male rats displayed a higher frequency of repetitive (marbles buried) and anxiolytic (open field center duration) behaviors compared to female rats during puberty and young adulthood. Further, aging increased anxiolytic and exploratory behaviors in both males and females. Exploratory behavior has been associated with increased center activity in open field assays [78, 79]. The testosterone [140, 141] and corticosterone levels [138, 139] observed in this study are consistent with the literature. Prior reports have shown similar levels of estradiol in males and females [142–145], and increased corticosterone levels in females compared to males [146, 147]. Since corticosterone has been linked with anxiety [148, 149], it is possible that these behavioral sex- and age differences may be related to the decreased plasma corticosterone in young adult males compared to young adult females.

The dopamine system has been associated with anxiolytic behaviors [150, 151] and repetitive behaviors [152]. We observed decreased hippocampal DAT in young adult females compared to young adult males. Along with this sex difference, we observed that young adult female rats had decreased hippocampal DAT, NR2A, and EGR-1 compared to pubertal female rats. There is not much information available on hippocampal DAT, as prior studies using Wistar, F344, and Sprague Dawley rats showed low DAT expression in the hippocampus [153–155]. Similarly, the data on sex differences in marble burying behavior is unclear. These marble burying studies were conducting using C57BL/6 mice, Wistar rats, and Sprague Dawley rats [156–159], in which there were no sex differences in mice and Wistar rats [156, 157] as compared to increased marble burying by Sprague Dawley female rats [158] and male mice [159]. Few studies on marble burying behavior incorporate both males and females [160], much less the impact of strain differences. Similarly, strain differences may mediate the equivocal findings on sex differences in center duration in an open field [78, 161, 162]. Prior studies that examined behaviors within the center of an open field range from no differences in center duration in Sprague Dawley rats [78] to increased center duration by Wistar female rats [161] and Long-Evans male rats [162]. Our study using Long-Evans rats is consistent with previous studies that showed increased center duration in Long-Evans rats that increased with age [162]. However, regardless of age, offspring in this study spent little time in the center of the open field, which could be an effect of arena size [161, 163].These strain differences may also affect the current “gold standard” valproate/valproic acid (VPA) animal model to study ASD in rodents [164], VPA may have greater efficacy inducing an ASD phenotype in males [165, 166], Behavioral phenotypes modelling ASD are typically observed only in male Wistar and Sprague Dawley rats [167–169]. However, the VPA phenotypes induced in Long-Evans are comparable between sexes [170, 171].

Limitations to using the late gestation CIH model to examine ASD include the inability to examine all behavioral phenotypes observed in ASD, such as interpersonal relationship formation. Although we did not assess environmental sensory responses and cognitive flexibility, these behaviors can be measured using behavior tests, such as acoustic startle response to detect dysfunctional fear generation following auditory stimuli [172] and cognitive function tests that include reversal learning, inhibitory learning, or set-shifting [173]. Vaginal smears for estrous cycles in female rats were not examined, so estradiol levels could not be compared to estrous status [174, 175]. Even so, high variability in estradiol levels was observed in both males and females, despite steroid hormone extraction. Although we found spatial memory dysfunction in response to late gestational CIH, future experiments on other memory domains (e.g., recollective memory) need to be conducted to determine the presence of memory impairments [176, 177]. Overall, the late gestational CIH model could be utilized as a novel preclinical model to explore the mechanisms underlying hypoxia-associated pregnancy complications on ASD risk, especially examining ASD-associated social function impairment in females.

## Figures and Tables

**Figure 4:** Anxiety-like behaviors. Females spent less time in the center of the open field compared to males, irrespective of age or gestational CIH (A, B). No effect of gestational CIH or sex was observed on center entries, regardless of age (C, D). Normalized by square-root transformation (C, D). Analyzed by Two-way ANOVA with Fisher’s LSD multiple comparisons tests. ANOVA significance indicated by: * = sex; Post-hoc significance indicated by: # versus normoxic female, ## versus normoxic male; ### versus CIH female; p≤0.05

**Table 1:** Expression of proteins associated with social and cognitive function within the dorsal hippocampus.

CA1		Normoxia			CIH		
			Puberty	Young Adult **		Puberty	Young Adult **
	Dopamine Transporter (DAT)	**F**	91.27 ± 29.25	71.10 ± 18.23 ^##^	**F**	104.42 ± 45.78	68.21 ± 31.17 ^##^
		**M** *	84.76 ± 26.75	90.48 ± 25.92 ^#^	**M** *	92.81 ± 13.74	105.94 ± 20.84 ^#^
			Puberty	Young Adult		Puberty	Young Adult
	Monoamine Oxidase A (MAO-A)	**F**	49.33 ± 13.76	52.03 ± 45.22	**F**	53.28 ± 14.38	43.49 ± 34.70
		**M**	58.68 ± 38.36	54.45 ± 42.02	**M**	55.20 ± 25.98	43.79 ± 51.97
			Puberty	Young Adult **		Puberty	Young Adult **
	NMDA Receptor 2A (NR2A)	**F**	75.11 ± 14.95	54.20 ± 19.37 ^##^	**F**	76.22 ± 27.92	51.17 ± 22.55 ^##^
		**M**	73.82 ± 13.56	70.74 ± 24.76	**M**	64.43 ± 18.25	60.33 ± 25.83
DG		Normoxia			CIH		
			Puberty	Young Adult		Puberty	Young Adult
	Doublecortin (DC)	**F**	99.27 ± 43.29	70.64 ± 45.29	**F**	79.45 ± 20.34	75.99 ± 46.04
		**M**	94.30 ± 40.15	77.03 ± 31.08	**M**	103.62 ± 31.93	85.80 ± 23.07
			Puberty	Young Adult **		Puberty	Young Adult **
	Early Growth Response 1 (EGR-1)	**F**	43.71 ± 36.80	6.20 ± 5.63 ^##^	**F**	37.94 ± 33.27	6.41 ± 7.49^##^
		**M**	43.00 ± 21.61	5.36 ± 4.42^##^	**M**	33.83 ± 9.68	8.48 ± 8.48 ^##^

All values represent percentage of Beta Actin expression. All values presented as mean ± SD. Analyzed by Two-way ANOVA with Fisher’s LSD multiple comparisons tests, n = 6/group. ANOVA significance indicated by:

* = sex

** = age;

Post-hoc significance indicated by:

# = versus female

## = versus puberty; p ≤ 0.05

**Table 2:** Plasma concentration of steroid hormones.

Corticosterone (ng/ml)	Normoxia			CIH		
		Puberty	Young Adult		Puberty	Young Adult
	**F**	307.46 ± 107.24	322.22 ± 63.38	**F**	457.58 ± 203.25 ***	246.75 ± 63.89 ^###^
	**M** **	168.94 ± 74.95 ^##^	168.32 ± 39.67 ^##^	**M** **	218.80 ± 92.28^##^	160.03 ± 57.16 ^##^
Estradiol (pg/ml)	Normoxia			CIH		
		Puberty	Young Adult		Puberty	Young Adult
	**F**	26.70 ± 4.11	55.15 ± 48.92	**F**	40.85 ± 26.79	67.80 ± 42.65
	**M**	42.74 ± 58.77	43.12 ± 30.50	**M**	39.99 ± 35.31	38.44 ± 18.53
Testosterone (ng/ml)	Normoxia			CIH		
		Puberty	Young Adult **		Puberty	Young Adult **
	**F**	0.51 ± 0.30	0.99 ± 0.48	**F**	0.70 ± 0.31	1.52 ± 0.95 ^###^
	**M** **	1.52 ± 0.67 ^##^	2.92 ± 1.08^##^, ^###^	**M** **	1.62 ± 0.62 ^##^	2.99 ± 0.69 ^##, ###^

All values presented as mean ± SD without normalization. All values normalized by square root transformation for analysis. Analyzed by Two-way ANOVA with Fisher’s LSD multiple comparisons tests. Corticosterone: n = 8–10/group; Estradiol: n = 5–9/group; Testosterone: n = 7–10/group. ANOVA significance indicated by:

* = CIH

** = sex or age

*** = interaction of age/CIH;

Post-hoc significance indicated by:

# = versus normoxia

## = versus female

### = versus puberty; p ≤ 0.05

## Data Availability

The datasets used and/or analyzed during the current study are available from the corresponding author upon reasonable request.

## Highlights

- Short term hypoxia during late gestation induced sex- and age-specific differences on social and cognitive function in offspring.
- Late gestational CIH effects on social and cognitive behaviors were mostly transient and present during puberty, except in females, which showed sustained social disengagement and suppressed corticosterone levels during young adulthood.

## Perspectives and significance

The diagnostic criteria for ASD requires deficits in social-emotional reciprocity, nonverbal communication/social interactions, interpersonal relationships, and the presence of restricted or repetitive patterns in behavior (e.g., stereotyped or repetitive movements/speech, inflexibility to routine, restricted/fixated interests, or abnormal sensory responses) [19]. In addition, ASD is associated with delays in speech production, cognitive impairments, hormonal dysregulation, and neurotransmitter dysfunctions [19, 20]. Behaviors associated with ASD diagnostic criteria were observed in our novel, late gestational CIH model (Supplemental Fig. 8). In response to late gestational CIH, we observed in this study and the associated study [9] fine motor dysfunction in male offspring, social function impairments in female offspring, repetitive behaviors or object fixation in female offspring, dysregulated circulating corticosterone levels in female offspring, and cognitive dysfunction in male offspring. Most social effects were only present in females exposed to gestational CIH, especially during the period of puberty. This is of concern as puberty is a critical period for social, endocrine, and cognitive maturation [24, 25], and girls are underdiagnosed for ASD [20–23]. Current American Academy of Pediatrics recommendations support ASD screening for all children up to their 24-month checkup [178]. Based on our findings, children from hypoxia-associated pregnancies should be screened for ASD throughout puberty. Importantly, the results from this study and the associated study [9] indicate that late pregnancy complications associated with hypoxia can have long-term effects on offspring that impact social, endocrine, and cognitive maturation.
